# Dietary Exposure to Acrylamide Has Negative Effects on the Gastrointestinal Tract: A Review

**DOI:** 10.3390/nu16132032

**Published:** 2024-06-26

**Authors:** Katarzyna Palus

**Affiliations:** Department of Clinical Physiology, Faculty of Veterinary Medicine, University of Warmia and Mazury in Olsztyn, Oczapowski Str. 13, 10-718 Olsztyn, Poland; katarzyna.palus@uwm.edu.pl; Tel.: +48-895234460

**Keywords:** acrylamide, cancer, enteric nervous system, gastrointestinal tract, gut microbiome, prevention strategies

## Abstract

Changing eating habits and an increase in consumption of thermally processed products have increased the risk of the harmful impact of chemical substances in food on consumer health. A 2002 report by the Swedish National Food Administration and scientists at Stockholm University on the formation of acrylamide in food products during frying, baking and grilling contributed to an increase in scientific interest in the subject. Acrylamide is a product of Maillard’s reaction, which is a non-enzymatic chemical reaction between reducing sugars and amino acids that takes place during thermal processing. The research conducted over the past 20 years has shown that consumption of acrylamide-containing products leads to disorders in human and animal organisms. The gastrointestinal tract is a complex regulatory system that determines the transport, grinding, and mixing of food, secretion of digestive juices, blood flow, growth and differentiation of tissues, and their protection. As the main route of acrylamide absorption from food, it is directly exposed to the harmful effects of acrylamide and its metabolite—glycidamide. Despite numerous studies on the effect of acrylamide on the digestive tract, no comprehensive analysis of the impact of this compound on the morphology, innervation, and secretory functions of the digestive system has been made so far. Acrylamide present in food products modifies the intestine morphology and the activity of intestinal enzymes, disrupts enteric nervous system function, affects the gut microbiome, and increases apoptosis, leading to gastrointestinal tract dysfunction. It has also been demonstrated that it interacts with other substances in food in the intestines, which increases its toxicity. This paper summarises the current knowledge of the impact of acrylamide on the gastrointestinal tract, including the enteric nervous system, and refers to strategies aimed at reducing its toxic effect.

## 1. Introduction

A 2002 report by the Swedish National Food Administration and scientists at Stockholm University on the formation of acrylamide in food products during frying, baking, and grilling contributed to an increase in scientific interest in the subject [1]. Acrylamide (ACR) is a product of Maillard’s reaction, which is a non-enzymatic chemical reaction between the carbonyl group in reducing sugars and the amine group in amino acids (especially asparagine) that takes place during thermal processing [2,3]. ACR has also been shown to form without reducing sugars in the acrolein pathway by enzymatic decarboxylation of asparagine or by thermal gluten decomposition [4,5,6]. The neurotoxic, genotoxic, carcinogenic, and reproductive toxicity of ACR have been confirmed in experiments on animal models and cell lines [7,8,9,10,11]. Considering the findings of studies on rodents, the International Agency for Research on Cancer (IARC) classified ACR as a probable human carcinogen (Group 2A) [12].

The toxicokinetics of ACR have been examined in rats, mice, and humans [13,14,15,16]. ACR is absorbed well in the gastrointestinal tract after oral administration [17]. The ACR absorption rate is species-dependent [18]. It was shown in a study with the human Caco-2 cell line that ACR intestinal transport is effected through passive diffusion, and it can be influenced slightly by pH and metabolic energy [19]. Upon absorption, ACR is distributed quickly to tissues—mainly to muscles, lungs, skin, alimentary tract, brain, testicles, and bone marrow [17,18,20,21,22]. It also passes through the placenta and into breast milk [23]. It largely undergoes reductive biotransformation by conjugation with glutathione (GSH), mediated by glutathione S-transferase [24]. After degradation and acetylation of the formed mercapturic acid, N-acetyl-S-(2-carbamoylethyl)-L-cysteine (AAMA) is excreted through urine [6,24]. Another ACR metabolic pathway (oxidative metabolic pathway) involves transformation to an epoxide derivative, i.e., glycidamide, mediated by cytochrome P450 (CYP2E1). The resulting glycidamide (also toxic) usually conjugates with glutathione, and the resulting conjugates are N-acetylated in subsequent stages, ultimately producing N-acetylcysteine derivatives, i.e., mercapturic acids, excreted through urine [24,25]. Glycidamide can also undergo enzymatic hydrolysis to form dihydropropanoamide [25]. It has been shown that although glycidamide formation is the main ACR transformation pathway in rodent organisms (60% in mice and 30% in rats), AAMA is the main ACR metabolite in humans [25,26]. Kopp et al. [26] suggest that it can explain a lower susceptibility to cancer in humans compared to rodents exposed to ACR in food because the carcinogenic properties of glycidamide have been well documented. ACR is completely eliminated from the body within 24 h—mainly through urine and—to a small extent—through faeces. However, the portion eliminated by each path is species dependent [24,26]. According to some reports, ACR metabolites persist longer in the mice’s skin and testicles [24].

The development of analytical methods, e.g., high-performance liquid chromatography (HPLC), gas chromatography (GC) with mass spectrometry (MS) detection, or in the selected ion monitoring (SIM) mode, or tandem mass spectrometry (MS/MS) in the multiple reaction mode (MRM) with isotope-labelled standards, enabled accurate determination of ACR content in individual food products, which made it possible to monitor the ACR level in ready products by food manufacturers [27,28]. According to the Joint Expert Committee for Food Additives (a UN Food and Agriculture Organization and World Health Organization advisory body), the main ACR sources in the diet contributing to the total ACR intake are chips (16–30%), potato crisps (6–46%), roasted coffee (13–39%), cakes, and cookies (10–20%), as well as various kinds of bread (10–30%) [28,29]. Since it is difficult to assess the body’s exposure to ACR, it is based on an analysis of specific biomarker concentrations, such as acrylamide and glycidamide adducts with DNA or haemoglobin. The level of adducts with haemoglobin is proportionate to the ACR dose in the body [29,30,31]. Using high-performance liquid chromatography with negative electrospray tandem mass spectrometry (HPLC/MS/MS), it was shown that there are large differences in acrylamide and glycidamide adducts with haemoglobin (ACR-Hb and GA-Hb adducts) between individuals and between subpopulations in Europe [32]. Epidemiological studies have shown that estimated ACR consumption in the diet ranges from 0.02 to 1.53 µg/kg b.w., and it is the highest in the European and American populations [33]. Infants, small children, and teenagers are particularly exposed to ACR in foods. Based on the European data, the main contributor to the total ACR exposure of infants was “Baby foods, other than processed cereal-based” followed by “Other products based on potatoes” and “Processed cereal-based baby foods”. The main contributor to the total exposure of toddlers, other children, and adolescents was “Potato fried products (except potato crisps and snacks)”, representing up to half the total exposure, followed by “Soft bread”, “Breakfast cereals”, “Biscuits, crackers, crisp bread”, “Other products based on cereals”, and “Other products based on potatoes” [28]. Relative to their body mass, their estimated daily intake of ACR is three times higher than that of adults [28,33]. The research conducted during the past 20 years has shown that consumption of ACR-containing products leads to disorders in human and animal organisms [6,7,8,9,10,11,21,22,27,33]. This effect is strictly dependent on the ACR dose, time of exposure to ACR, and the species of animals used in the experiment.

The gastrointestinal tract is a complex regulatory system that determines the transport, grinding, and mixing of food, secretion of digestive juices, blood flow, growth and differentiation of tissues, and their protection. The functions of the digestive system are regulated at an internal and external level. The unique internal control system, which consists of the enteric nervous system (ENS), the intestinal endocrine system, and the intestinal immune system, allows digestive functions to quickly adapt to changing local conditions. As the place of first contact with chemicals contained in food, it also plays an important protective role. Disorders in the functioning of the digestive tract lead to many health consequences and may have a negative impact on the entire body [34]. The gastrointestinal tract, as the main route of ACR absorption from food, is directly exposed to the harmful effects of ACR and its metabolite—glycidamide [17]. Moreover, the latest studies suggest that emulsifiers, e.g., polyoxyethylene sorbitan, being themselves of low toxicity, increase cell permeability and, in consequence, the absorption of chemical substances present in food, including acrylamide [35]. On the other hand, ACR has been shown to bind to chicken egg albumin while cooking or in the intestinal lumen, which lowers its bioavailability [19]. 

Despite numerous studies on the effect of ACR on the digestive tract, no comprehensive analysis of the impact of this compound on the morphology, innervation, and secretory functions of the digestive system has been made so far. According to the findings of studies conducted to date, oral exposure to ACR leads to the modification of intestine morphology and the activity of intestinal enzymes, as well as disruption of enteric nervous system function [36,37,38,39,40]. It also affects the gut microbiome and increases apoptosis, leading to the alimentary tract dysfunction [24,37,41]. In order to ensure food safety and limit the ACR amount in food, scientists are trying to develop methods of limiting its absorption and bioavailability by using various food additives or microorganisms [6,19,24,37,38]. This paper summarises the current knowledge of the ACR impact on the alimentary tract, including the enteric nervous system, and refers to strategies aimed at reducing its toxic effect. 

## 2. Impact of Acrylamide on the Gastrointestinal Tract Morphology and the Functional Features of the Intestine

As the first point of contact between ACR in foods and the body, the gastrointestinal tract is especially vulnerable to its harmful effects (summarized in Table 1). The findings of earlier studies showed that male Sprague-Dawley albino rats receiving ACR at 30 mg/kg b.w./day for four weeks had gastric mucosa erosions, considerably reduced mucosa thickness, accompanied by a vast inflammatory infiltration. Moreover, an increased expression of caspase-3 (a marker of apoptosis) was observed, as well as a decreased expression of the epidermal growth factor receptor (EPGR), which plays an important role in mechanisms of proliferation, differentiation, and repair of mucosa cells [41]. Other research on rats has shown that ACR disrupts an antioxidative protection system and increases lipid peroxidation in the gastric and hepatic mucosa [42]. The extent to which the mucosa is damaged is directly correlated with the dose and time of exposure to ACR. Long-term exposure to ACR results in gastric bleeding episodes, which are a consequence of vast lesions in the gastric mucosa [43].

The cytotoxic effect of ACR on the intestines was confirmed in studies on animal models and on cell lines [37,38,44,45,46,47]. Intoxication with ACR in BALB/c mice resulted in changes in the histomorphometry of the jejunum wall, such as a decrease in the length of intestinal villi, the depth of crypts, the number of active crypts, and the size of the intestine absorption surface. ACR also considerably reduced the number of proliferating cells, while the number of apoptotic cells increased [37]. Further studies demonstrated that functional disorders of the small intestine, whose main function is to absorb nutrients and pass them on to the circulation, can be a consequence of damage to the intestinal mucosa as a result of exposure to reactive oxygen species (ROS) [10,44]. A growing number of reports confirm that oxidative stress is one of the main mechanisms of the harmful effect of ACR on the intestines. ACR consumption (25 mg/kg for three weeks) reduced the glutathione (GSH) level and the total antioxidative status (TAS) in the intestines and increased the activity of superoxide dismutase (SOD) and catalase (CAT), as well as the total oxidation state (TOS) and the level of malondialdehyde (MDA) in the ileum and colon of Wistar albino rats, leading to modifications in the histological structure. Degeneration and shortening of intestinal villi were observed, as well as damage to the ileum mucosa, inflammatory cell infiltration and degeneration of the surface epithelium and Liberkühn crypts in the colon [38]. This was also confirmed in other research, where intoxication with ACR resulted in an elevated MDA level, a considerable decrease in the GSH level and glutathione S-transferase (GST) activity in the small intestine and liver in rats. Oxidative stress induced by ACR caused damage to the intestinal villi structure, reducing the cell density in the lamina propria and damaging the intestinal epithelium. Strong vacuolisation and infiltration of eosinophils were observed in the liver [45]. This is consistent with the findings obtained on human HepG2 and Caco-2 cell lines, where exposure to ACR led to dramatic growth in the production of ROS and superoxide anion radicals, a decrease in the mitochondrial membrane potential (MMP), mitochondrial membrane lipid peroxidation, exhaustion of GSH, and a lowering of the enzymatic activity of SOD and CAT [46,47]. 

The intestinal barrier is the main line of the body’s defence against harmful substances in the intestinal lumen. Tight junctions (TJs) consist of clusters of transmembrane proteins (e.g., zonula occludens, occludin, and claudins 1–5) that play a key role in the intestinal barrier, preventing infiltration of intestinal bacteria and other chemical compounds from the intestinal lumen to the systemic circulation [48]. There is proof that ACR affects the integrity of tight junctions, leading to barrier dysfunction and intestine damage [48,49,50]. Research with the rat small intestine epithelium cell line (IEC-6 cells) showed that exposure to ACR decreased cell viability, increased intercellular permeability and release of lactic dehydrogenase, and destroyed tight junctions [48,51]. Cytotoxicity of ACR and intestinal barrier damage have also been confirmed in human Caco-2 cell lines [52]. According to the findings of recent studies, by reducing the tight junction protein expression, ACR disrupts the intestine permeability, which weakens the epithelial barrier of the intestines, thereby increasing the intensity of food allergy symptoms [53]. 

ACR has also been shown to cross the placenta and to have an adverse impact on the development of the gastrointestinal tract in the offspring of mothers exposed to ACR in pregnancy [36,40,54]. Exposure to ACR of Wistar rats at 3 mg/kg b.w./day for 0, 5, 10, and 15 days disrupts the structural and functional development of the small intestine in weaned offspring, depending on the time and the intestine segment. It has been shown that intrauterine intoxication with ACR changes the small intestine morphology (a decrease in the length, thickness and volume of the villi, of the depth of crypts, an increase in the villi height to crypt depth ratio, a decrease in the crypt and mucosal membrane thickness), disrupts the expression of proteins forming adhering and tight junctions (E-adherin, occludin), increases the activity of intestinal enzymes, and increases apoptosis [36]. Similar changes have been described in the histomorphometry of the small intestine in guinea pigs exposed to ACR during the foetal period of the last 35 days of gestation. Treatment with ACR in the mother (at 3 mg/kg b.w./day) had a negative impact on the integrity and innervation of the small intestine wall and its absorbing surface [54]. Early changes as a consequence of pre- or postnatal exposure to toxins can be a sign of a disruption of metabolic processes. A study conducted by Walden et al. [40] demonstrated that rats exposed to ACR during the foetal and lactation periods have a significantly modified level of intestinal enzymes (acid phosphatase, alkaline phosphatase, beta-glucuronidase, citrate synthase, and lactic dehydrogenase) at early growth phases. Further studies showed that intrauterine exposure to ACR had an impact on the functional development of the rat intestine through a change in the immunolocalization of leptin, ghrelin, and their receptors [36].

Being one of the elements of mucosa-associated lymphoid tissue (MALT), gut-associated lymphoid tissue (GALT) is a barrier protecting the body from infections and systemic inflammation but also plays a role in recognising, intercepting, and fighting harmful pathogens ingested with food [55]. Immune system cells form closely clustered lumps and ileal Peyer’s patches (IPP) in the lymphatic tissue, as well as single lymphatic lumps and clusters of plasmatic cells producing IgA antibodies [55,56]. The modulating effect of ACR on the systemic immune system has been well researched [57]. Since the intestines are the first point of contact with ACR in food, it could be expected that ACR would have an impact on the local mucous membrane immune system. Yener et al. [56] report that ACR at large doses has a cytotoxic effect on IPP in Sprague-Dawley rats. The lymphoid follicles of the IPPs were markedly reduced in size, and the germinal centres regressed. Moreover, ACR reduces the population of alpha-naphthyl acetate esterase (ANAE)-positive lymphocytes, both in peripheral blood and in IPP. Furthermore, research conducted on a pig model showed that supplementation with ACR leads to an increase in the level of pro-inflammatory cytokines (interleukin 1 β (IL-1β), IL-6, and tumour necrosis factor-α (TNF-α)) synthesised in the IPP in the ileum [39].

**Table 1:** The impact of ACR on the GI tract morphology and function.

GI Tract Segment	Species	Dose of ACR and Time of Exposure	The Result of Study	References
stomach	rats	30 mg/kg of b.w./day for 4 weeks	gastric mucosa erosions, reduced mucosa thickness, inflammatory infiltration, apoptosis	[41]
stomach, liver	rats	0.05% in drinking water for 40 days	increased lipid peroxidation (MDA level), decreased level of GSH	[42]
jejunum	mice	0.5 mg/kg of b.w./day for 90 days	a decrease in the length of intestinal villi, the depth of crypts, the number of active crypts and the size of the intestine absorption surface; a reduced number of proliferating cells, an increased number of apoptotic cells	[37]
ileum, colon	rats	25 mg/kg of b.w./day for 21 days	degeneration and shortening of intestinal villi, damage of ileum mucosa, inflammatory cell infiltration; degeneration of surface epithelium and Liberkühn crypts in the colon	[38]
ileum	rats	0, 30, 45 and 60 mg/kg of b.w./day for 5 days	decreased ANAE-positive peripheral blood lymphocyte levels in a dose dependent manner	[56]
ileum	rats	0, 125, 150, and 175 mg/kg of b.w in a single dose	reduced lymphoid follicles of the ileal Peyer’s patches (IPPs), regressed germinal centres (GCs), ANAE-positive lymphocyte depletion in IPPs	[56]
ileum	pig	0.5 and 5 μg/kg of b.w./day for 28 days	an increase in the level of pro-inflammatory cytokines (interleukin 1 β (IL-1β), IL-6 and tumour necrosis factor-α (TNF-α)) synthesised in the IPP	[39]
small intestine, liver	rats	25 mg/kg of b.w./day for 21 days	damage to the intestinal villi structure, the reduced cell density in the lamina propria, damaged intestinal epithelium, strong vacuolisation and infiltration of eosinophils in the liver	[45]
small intestine	mice	0, 10 and 100 mg/kg of b.w./day for 28 days	reduced the expression of intestinal tight junction proteins, disrupted the permeability of the intestine, which impaired the intestinal epithelial barrier	[53]
small intestine	rats	3 mg/kg of b.w./day for 0, 5, 10 and 15 days in utero	a decrease in the length, thickness and volume of the villi and the depth of crypts, an increase in the villi height to crypt depth ratio, a decrease in the crypt and mucosal membrane thickness, decreased expression of tight junction proteins (E-adherin, occludin), decreased activity of intestinal enzymes, increased apoptosis	[36]
small intestine	guinea pigs	3 mg/kg of b.w./day for last 35 days of gestation	increased thickness of myenteron and submucosa, mucosa fractal dimension and the depth of crypts in the duodenum, increased the number of total, divided and inactive crypt, and the number of damaged villi in the duodenum and jejunum, increased the total villi number in the jejunum, the decrease of villi epithelium thickness and active crypt number in the jejunum, decreased goblet cells number and intact villi number in the duodenum and mucosa thickness and crypts width in the jejunum, higher size of nerve plexuses in duodenum, decreased expression of cadherin, increased apoptotic cell number	[54]
small intestine	rats	20 mg/kg of b.w./day for last 10 days of gestation and during lactation	disturbances in intestinal enzymes activities (acid phosphatase, alkaline phosphatase, beta-glucuronidase, citrate synthase and lactic dehydrogenase)	[40]
Caco-2 cells	human	2.5 mM ACR for 24 h	decreased cell viability, mitochondrial membrane potential (MMP) collapse	[46]
Caco-2 cells	human	7.5 mmol/L ACR for 4–20 h	decreased transepithelial electrical resistance (TEER) value; mitochondrial membrane potential (MMP) collapse, decreased FITC-dextran 4 kDa permeability, increased apoptosis, increased lactic dehydrogenase (LDH) release, decreased expression of claudin-1, occludin and zonula occludens-1	[52]
HepG2 cells	human	2.5 mM ACR for 24 h	decreased cell viability, mitochondrial membrane potential (MMP) collapse	[47]
IEC-6 cells	rats	1.25–10 mmol/L for 24 h	decreased cell viability, increased intercellular permeability and release of lactic dehydrogenase, destroyed tight junctions	[48]
IEC-6 cells	rats	2.5 mM ACR for 24 h	decreased cell viability, decreased transepithelial resistance (TEER) value of the cell monolayer, suppressed protein expression of the tight junction proteins (occludin, claudin-1, and zonula occludens-1)	[51]

## 3. Acrylamide Interaction with Gut Microbiome 

**Figure 1:**
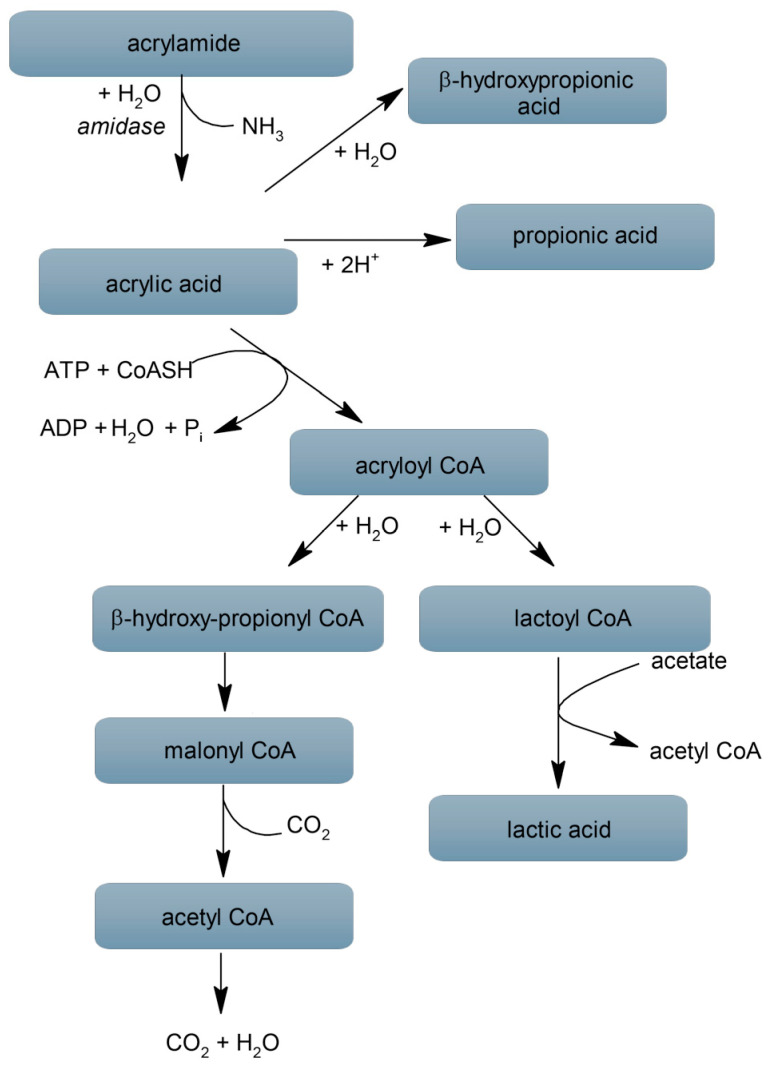
Decomposition of ACR by amidases and further transformations of acrylic acid (due to [24,65]).

Bacteria inhabiting the alimentary tract, which form the gut microbiome, take part not only in digestion but also in supporting homeostasis of the whole body, affecting immunity, metabolism, the synthesis of many chemicals, and immune protection. They are also involved in the decomposition of exfoliated epithelial cells, bile components, some medicines, and potential carcinogens [58]. Although the impact of ACR on the gut microflora has not been sufficiently researched, it has been shown that various microorganisms can degrade ACR and its metabolites [24]. Previous studies have shown that various strains of bacteria in a very wide range of concentrations (from 1 to 40 mM) (data collected in Table 2) catalyze the ACR hydrolysis to ammonia and acrylic acid through the production of amidases [59,60,61,62,63,64,65,66,67,68,69,70,71,72,73] (Figure 1). Among the bacteria that inhabit the human body, it has been shown that Helicobacter pylori, Escherichia coli, Enterococcus faecalis, and Bacillus clausii secrete amidase [24,66,74,75]. Helicobacter pylori, causing gastritis and peptic ulcer disease, uses the activity of amidase and urease to survive in the unfavourable environment of the stomach [76]. Whereas Escherichia coli synthesises penicillin amidase catalysing the decomposition of penicillin to carboxylic acid and 6-aminopenicillanic acid, which leads to a reduction in its antibacterial properties [77]. The synthesis of amidases by bacteria naturally occurring in the gastrointestinal tract may lead to the degradation of ACR and reduce its harmful effects on humans. However, further research confirming that these bacteria are able to degrade ACR in human intestine is necessary. Moreover, lactic acid bacteria were shown to have the ability to reduce the ACR level in food products by reducing the amount of simple sugars. These properties can be used to reduce the ACR content in the diet [24,78]. Further, studies on mice have shown that supplementation with Bifidobacterium reduced ACR-induced toxic DNA damage in the colon and liver [79]. Research by Sequer et al. [80] on adult volunteers showed that Maillard reaction products (MRP) modified the population size of the bacteria comprising the gut microflora in humans, depending on the chemical structure and amount of individual MRPs in the diet. In rats receiving feed rich in MRPs, a reduction in the general bacterial population, especially the number of lactobacilli, was observed [80]. 

## 4. Impact of Acrylamide on the Enteric Nervous System

ACR is well known for its neurotoxic properties, as its toxic effect on the nervous tissue has been demonstrated both in animal models and by observation of exposure in humans [7,81]. Long-term exposure to ACR leads to nerve fibre damage in the central and peripheral nervous system, nerve conduction disorders (fast axonal transport), and inhibition of neurotransmitter release, which manifests itself clinically as weakening of the limbs, tingling, convulsions, and ataxia [7,82]. By binding and inhibiting the motor protein, i.e., kinesin, ACR inhibits fast axonal transport, both anterograde and retrograde [83]. It has been shown that by inhibiting creatine kinase, ACR decreases the ATP level in nerve cells, leading to their death, and its high affiliation to cysteine-rich proteins results in inhibition of neurotransmitter release from the presynaptic membrane [82]. Moreover, Faria et al. [84] demonstrated that ACR disrupts the synthesis of proteins essential to the function of a nerve cell in zebrafish and considerably reduces the level of genes associated with regeneration.

**Figure 2:**
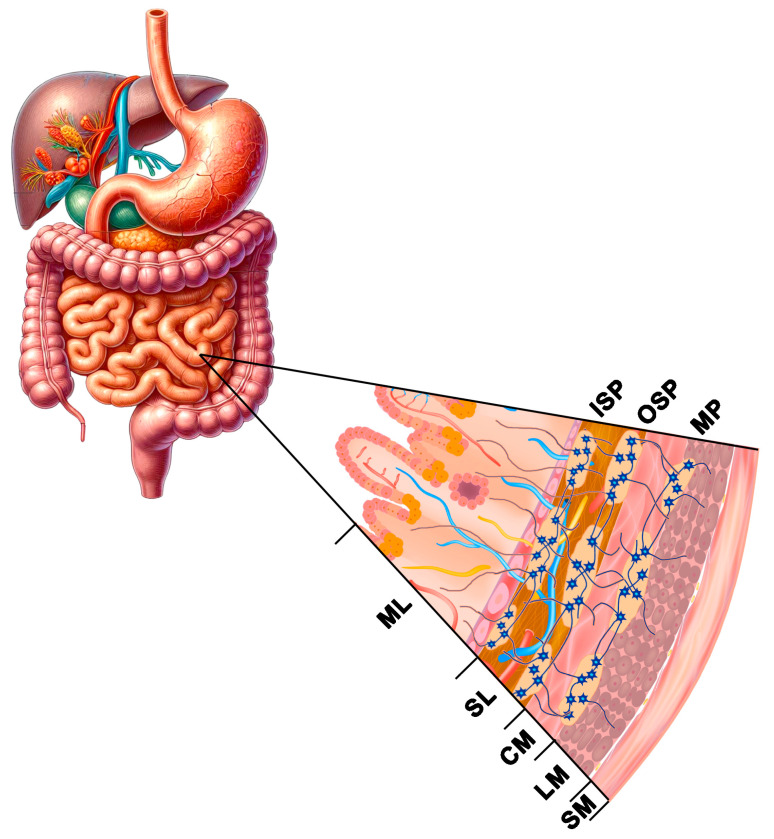
Diagram illustrating the enteric nervous system in the human small intestine. Intamural plexuses: MP—myenteric plexus, OSP—outer submucous plexus, ISP—inner submucous plexus. Parts of the intestinal wall: SM—serosal membrane, LM—longitudinal muscle layer, CM—circular muscle layer, SL—submucosal layer, ML—mucosal layer.

The nerve control of the gastrointestinal tract function is effected by extrinsic innervation (central regulation by sympathetic and parasympathetic neurons) and by intrinsic innervation—intramural neurons, which make up the enteric nervous system (ENS) [85,86]. The ENS plays an essential role in the regulation of physiological processes in the intestines, where over 200 million neurons divided into functional subclasses (motor neurons, interneurons, and sensory neurons) control motility, intestinal secretion, transmucosal water flow, and local blood flow [85]. The uniqueness of the ENS is associated with high independence in the regulation of processes in the intestines, effected by a large group of neurotransmitters and/or neuromodulators, because of which it is called “the gut-brain” [86]. The ENS is located in the gastrointestinal wall from the oesophagus to the rectum inner sphincter, where it forms intestinal plexuses connected with a dense network of nerve fibres [39,85,86]. Depending on its location on the wall cross-section, one can distinguish the myenteric (Auerbach’s) plexus between the circular and longitudinal muscularis layer and the submucous (Meissner’s) plexus situated in the mucous membrane. There are interspecies differences in the structure and spatial organisation of the ENS. The myenteric plexus and single submucosal plexuses are mainly present in all species in the oesophagus and the stomach. Two types of plexuses (myenteric and submucous) are present in the rodents’ intestines, whereas large mammals and humans have myenteric plexuses and two types of submucous plexuses: the outer submucous plexus (OSP), situated near the outer layer of circular muscles, and the inner submucous plexus (ISP), situated near the lamina propria of the mucous membrane [39,85,86,87] (Figure 2). It has been demonstrated in many studies that the ENS exhibits high plasticity in the sense of adaptability, both morphological and functional, in response to inflammatory factors, metabolic disorders, and toxins in food, and it is the first line of defence against harmful factors present in the intestinal lumen [85,87].

Previous studies have shown that ACR in food has a significant impact on the ENS, which is presented below and summarised in Table 3. Long-term administration of ACR in drinking water to mice resulted in morphological changes in intramural nerve plexuses, e.g., an increase in their cross-sections and density of nerve fibres [37]. Further, Tomaszewska et al. [54] report that newborn guinea pigs exposed to ACR in utero have nerve plexuses in the duodenum that are 2.5 times larger than their peers in the control group. A considerable increase in the plexus circumference and diameter has been observed in submucosal plexuses and slightly smaller in myenteric plexuses, which can disrupt secretory activity and intestinal motility in later life. 

Given that the ENS demonstrates a high level of neuronal plasticity in response to inflammatory factors, neuron damage, and food toxins, manifesting itself as changes in the immunohistochemical phenotype of neurons, the impact of ACR on the neurochemical and neurotransmitter content in intestinal neurons was determined. Belai and Burnstock [88] used HPLC and the immunoenzymatic assay to show that intoxication with ACR leads to a decrease in the tissue content of noradrenaline, a decrease in immunoreactivity and the tissue content of calcitonin gene-related peptide (CGRP), an increase in vasoactive intestinal polypeptide (VIP), and the absence of an impact on the level of neuropeptide Y (NPY) and substance P (SP) in the myenteric plexus in the rat ileum. Exposure to ACR resulted in a decrease in the tissue content and immunoreactivity of CGRP and an increase in VIP and NPY. No changes in the SP content or immunoreactivity were observed.

Further studies with a domestic pig model showed that a 4-week supplementation of ACR considerably altered the chemical coding of ENS neurons. The changes depended on the dose and the gastrointestinal tract section under investigation [39,86,89,90,91,92,93,94,95]. In the stomach, low-dose ACR supplementation corresponding to a tolerable daily intake (TDI) (0.5 µg/kg b.w./day) caused an increase in the population of neurons immunoreactive to galanin (GAL), cocaine- and amphetamine-regulated transcript peptide (CART), vesicular acetylcholine transporter (VAChT), VIP, and SP in submucous plexuses in the corpus. However, it did not have an impact on neuronal nitric oxide synthase (nNOS)-immunopositive neurons [89,90,91]. Exposure to a larger ACR dose (5 μg/kg b.w./day) resulted in an increase in the number of GAL-, CART-, VAChT-, VIP-, SP-, and nNOS- immunopositive neurons in submucous plexuses in the corpus of the stomach. In turn, in myenteric plexuses, ACR at a low dose increased the number of GAL-, nNOS-, and VIP-immunoreactive (IR) neurons in the cardia, corpus, and pylorus; VAChT- and SP-IR in the corpus and pylorus; and in the case of CART, the changes were significant only in the corpus and CGRP only in the pylorus. However, in all examined sections of the stomach (cardia, corpus, pylorus), an increase in the number of myenteric plexuses neurons immunopositive for all tested neuroactive substances (GAL, CART, VAChT, VIP, SP, CGRP, nNOS) was noted [89,90,91,92].

In the duodenum, oral supplementation of ACR increased the number of intramural pituitary adenylate cyclase-activating polypeptide (PACAP)-, CART-, CGRP-, VAChT-, VIP-, nNOS-, GAL-, and SP-IR neurons [86,92,93,94]. However, the intensity of the changes depended on the type of analysed intestinal plexus and the neuroactive substance under examination. An increased population of PACAP-, CART-, CGRP-, VAChT-, GAL-, and SP-positive neurons in the myenteric plexuses was observed as a consequence of the administration of small ACR doses to pigs. However, this did not have an impact on the number of VIP- or nNOS- IR neurons. Only an increase in the number of VAChT-, GAL-, and SP-positive neurons in the OSP was observed in the group which received a small ACR dose. On the other hand, a small dose of ACR increased the population of CGRP-, VAChT-, VIP-, GAL-, and SP-IR neurons in the ISP. Exposure to large ACR doses resulted in an increase in the number of immunoreactive neurons with respect to all the neurotransmitters under study in all three types of enteric plexuses (MP, OSP, and ISP) [86,92,93,94]. 

In the jejunum, small ACR doses caused the greatest changes in the chemical coding of the myenteric plexus neurons. Increased immunoreactivity with respect to PACAP, CART, CGRP, VAChT, GAL, and SP was observed [86,92,93,94,95]. The increase in the OSP was significant only in the case of PACAP. When it comes to the ISP, an increase in the number of PACAP-, VIP-, and GAL-IR and a decrease in the nNOS-IR ones were demonstrated. An increase in the population of PACAP-, CART-, CGRP-, VAChT-, VIP-, GAL-, and SP-IR and a decrease in that of nNOS-IR neurons were visible in all the intramural plexuses in the case of a large dose of ACR [86,92,93,94,95].

Further, an increase in the number of CART-, CGRP-, VIP-, GAL-, and SP immunopositive and a decrease in the number of nNOS-positive myenteric neurons in the ileum were observed in the group receiving a small ACR dose. Further, a small ACR dose brought about an increase in immunoreactivity with respect to VAChT, VIP, and SP and a decrease in the number of nNOS-IR neurons in the OSP. In turn, the largest increase in the ISP was observed in the case of CGRP, VAChT, VIP, and GAL. Similar to other parts of the small intestine, exposure to high doses of ACR caused changes in the chemical coding of intramural neurons in both the myometrium and mucosa. ACR increased the number of PACAP-, CART-, CGRP-, VAChT-, VIP-, GAL-, and SP-IR neurons and decreased the number of nNOS-positive ones [39,86,92,93,94]. The data suggest that the neuroactive substances under study can play a significant role in acrylamide-induced neuroplasticity and be an important line of defence for ENS neurons against the harmful effects of ACR. Although the mechanism of the observed changes is not known, the existing knowledge of the ACR impact on the nervous system suggests that it can also have a neurotoxic effect on ENS neurons.

## 5. Potential Carcinogenic Effect 

Animal studies have demonstrated beyond doubt that ACR is a carcinogen for rodents, causing cancers of many organs: the thyroid gland, lungs, testicles, mammary gland, liver, brain, and skin [96,97,98]. DNA adduction by glycidamide and resulting mutagenesis are believed to play a key role in the development of ACR-induced cancers [26,99]. Glycidamide has been shown to be genotoxic both in vivo and in vitro [99,100]. However, this mechanism has been confirmed conclusively only in the case of mammary gland and testicle cancer in rats [101]. There are also other, non-genotoxic theories concerning the mechanisms of ACR carcinogenic properties, such as exhaustion of the GSH supply, leading—through oxidative stress—to changes in gene expression, the hormone effect in cancer formation in hormone-dependent organs, or ACR inhibiting the mitotic/meiotic motor protein, leading to defects in cell division [6,102]. 

The gastrointestinal tract, which is the first point of contact with ACR in food, can be potentially susceptible to its carcinogenic effect. The findings of studies on the impact of dietary exposure to ACR on the development of various cancers in the gastrointestinal tract are contradictory. Several experiments with rodents have been performed so far (data in Table 4). Zhang [103] studied the impact of simultaneous exposure to ACR and corn oil on the development of colorectal cancer in Sprague-Dawley rats. It was shown that long-term (48 weeks) simultaneous exposure to ACR and corn oil in the diet induces colorectal cancer development through increased proliferation and inhibition of mitochondria-dependent apoptosis mediated by the p53 tumour suppressor gene. Further studies on rats showed exposure to ACR to induce pre-cancer changes in the colorectal mucosa by Wnt/beta-catenin signalling, and the presence of corn oil and beef in the diet increased, while the addition of olive oil and fish oil reduced the changes induced by ACR [104]. A study conducted by Raju et al. [105] showed that ACR administered orally at 50 mg/kg b.w. for eight weeks did not induce pre-cancer lesions in rat colons, regardless of the fat content of the feed. Further research showed that ACR alone at doses corresponding to actual levels in food does not induce cancer in rats, but it can intensify azoxymethane-induced cancers when administered at larger doses [106]. Additionally, in order to assess whether ACR has an impact on the development of colorectal adenocarcinoma, mice had cancer xenografts performed from human colorectal adenocarcinoma HT-29, and they were given feed with ACR for four weeks. Exposure to ACR in feed did not have an impact on the cancer progression [106]. The latest studies on mice that were given ACR orally (0.1 mg/kg/day) for four weeks demonstrated changes in the expression of genes in the colon. Increased expression of genes involved in RNA metabolism, the processing and formation of ribosome subunits, as well as translation and protein metabolism, was observed in the group receiving ACR, which correlated positively with hyperexpression of the genes in samples of human colorectal adenocarcinoma [107]. The research findings suggest that ACR can be involved in the induction of gastrointestinal cancer in rodents, depending on the dose, exposure time, and co-occurrence of various potentially carcinogenic environmental factors.

Since ACR is a potential animal carcinogen, one should note that it may have a similar effect on the human organism and can pose the threat of cancer development. Numerous epidemiological studies conducted so far and discussed in a report by Basaran et al. [98] have shown that dietetic exposure to ACR can be linked to an increase in the risk of gastrointestinal cancer in people. However, the risk assessment is difficult, and various results were obtained depending on the research method (prospective cohort study, case-cohort studies, case-control studies, meta-analysis studies). It has only been confirmed so far that ACR increases the risk of oesophageal cancer, particularly in obese people and non-smokers [108,109]. A cohort study conducted on elderly people in China showed dietary exposure to ACR could potentially increase the overall mortality caused by gastrointestinal cancers [110]. Further studies have confirmed that it has no impact on the development of gastric, colorectal, hepatic, or pancreatic cancer [98,111]. However, the latest meta-analysis performed by Zhang et al. [112] showed consumption of foods rich in ACR increases the risk of gastric cancer both in Asian and non-Asian populations. However, these studies have some limitations. It is not possible to exactly determine the ACR dose that was consumed—it is based on an assessment of the ACR content of individual food products in a geographic region. The amount of consumed products is based on the questionnaires completed by the study participants. An assessment of exposure to ACR by measuring the levels of ACR adducts with haemoglobin determines only exposure within the past three months, and research is usually conducted over many years. It is also worth mentioning that food is not the only source of human exposure to ACR. People are potentially exposed to ACR in tobacco smoke (cigarette and e-cigarette tobacco smoke) or chemical products (occupational exposure). ACR is mainly used for the synthesis of polyacrylamides used in sewage treatment processes, paper production, ore processing, the production of vinyl polymers, and as a sealant in the construction of dams and tunnels, while polyacrylamide gel is used in the electrophoresis process commonly used in many laboratories. Humans can be exposed to ACR through oral, dermal, and inhalational routes [113,114]. This makes it difficult to assess real human exposure to this substance. Moreover, the lower susceptibility of humans to ACR-induced cancers is probably attributable to the different metabolism of ACR and the dominance of reductive biotransformation, ultimately leading to the formation of AAMA, and a small proportion of oxidative processes, leading to the formation of glycidamide [26]. Further studies in this regard are necessary to confirm/exclude the carcinogenic effect of ACR on the human organism.

## 6. Strategies for Decreasing ACR Levels in Food Products and Limiting Its Toxic Effect on the Gastrointestinal Tract

Due to the adverse effect of ACR in food products on the human organism, the food industry and regulatory bodies—both in Europe and in the USA—took measures to decrease the ACR levels in food, according to the ALARA (“As Low As Reasonably Achievable”) principle. The “Toolbox” for acrylamide, developed by the Confederation of the Food and Drink Industries of the EU (French: Confédération des Industries Agro-Alimentaires de l’UE, CIAA), describes the available strategies aimed at reducing ACR content of food [115]. They are mainly concerned with cereals and potato products, which are the major sources of exposure to ACR [6]. The most important of them include the choice of potato cultivars or cereal species containing low levels of ACR precursors (mainly asparagine), the addition of asparaginase (an enzyme reducing the asparagine level by up to 90%), replacing reducing sugars with sucrose and ammonium bicarbonate with sodium bicarbonate, changes in the production technology (change of duration, temperature of frying or baking, changing the type of oven, prolonged fermentation), and the removal/binding of ACR in a finished product by chromatography, evaporation, polymerisation, or by reaction with other food products (e.g., food protein) [6,19,115,116]. However, one has to note that the large-scale application of these methods has some limitations. The first of them is an increase in production costs (a high price of asparaginase, more expensive raw materials, and a longer production time). There is also no proof that the simultaneous application of several methods will contribute to a larger decrease in the ACR level in the finished product than a single strategy. Interactions between different agents are also unknown, and it is not certain whether a decrease in the ACR level will result in the formation of other potentially harmful substances. The available data suggest that strategies for decreasing the ACR levels in cereal products often lead to the formation of another impurity—5-hydroxymethylfurfural (HMF) [117]. Moreover, these strategies often have a negative impact on the organoleptic characteristics of the finished product (brown crust or crispiness), which may decrease consumers’ interest in a less organoleptically attractive product despite a lower ACR level [6].

The next research trend concerns decreasing the in vivo toxicity of ACR. Experimental studies conducted so far, both on cell lines and on animal models, have demonstrated that the toxic effect of ACR on the gastrointestinal tract can be limited by using food additives [37,38,41,45,46,47,48,49,50,51,52,118,119,120,121,122,123,124]. Dobrowolski et al. [37] showed that a 2% addition of warmed or raw potato fibre to a mouse diet eliminates the negative ACR impact on the histological structure, innervation of the small intestine wall, and absorptive function of the mucous membrane. Other researchers report that crocin exhibits a protective effect against ACR-induced small and large intestine damage in rats, thereby reducing histological damage [38]. Furthermore, the addition of rosemary to feed reduced acrylamide-induced inflammation, cell apoptosis, and oxidative stress and accelerated the healing of the gastric mucosa in adult male albino rats [41]. The latest research on BALB/c mice confirmed that fermented polysaccharides from Hericium erinaceus reinforce the intestinal barrier function by increasing the level of cyclophosphamide, expression of occludin, zonula occludens-1, zonula occludens-2, claudin-3, claudin-4, and mucin, and a change in the gut microflora composition. It has also been shown that fermented polysaccharides protect IEC-6 cells against damage caused by ACR by increasing the expression of tight junctions and mucins [49]. Further, Fan et al. [51] demonstrated that natural flavonols (quercetin and myricetin) can alleviate acrylamide-induced IEC-6 cell damage, which results in higher cell viability, lower lactate dehydrogenase, and decreased formation of reactive oxygen species. Both substances have been shown to decrease para-cellular permeability and increase the expression of three tight junction proteins (occludin, claudin-1, and zonula occludens-1), but their beneficial effect is reduced considerably if the compounds are subjected to thermal treatment. The oligochitosan-glycated caseinate digest can also provide better protection against ACR-induced damage to the IEC-6 cell barrier. Both digestion products increase the expression of ZO-1, occludin, and claudin-1 [48]. A study conducted by Yan et al. [52] showed that procyanidin A1 and the products of its digestion considerably increased the transepithelial electrical resistance (TEER), decreased FITC-dextran 4 kDa (FITC-4 kDa) permeability, apoptosis, and lactate dehydrogenase (LDH) secretion, and increased the expression of claudine-1, occludin, and zonula occludens-1 in a Caco-2 monolayer cell membrane incubated with ACR. They have also been shown to inhibit the phosphorylation of mitogen-activated protein kinase (MAPK) and myosin light-chain kinase (MLCK) signalling pathways, thereby maintaining the normal functions of the intestinal barrier. 

Previous studies have shown oxidative stress to be one of the main mechanisms responsible for ACR toxicity [118,119,120]. Therefore, attempts have been made to employ antioxidative compounds to restrict the toxic effect of ACR on the gastrointestinal tract. Chen et al. [121] reported that miricitrin, a natural dietary compound, has antioxidative properties and protects human Caco-2 cells against the toxic ACR effect in a concentration-dependent way by inhibiting the production of reactive oxygen species (ROS). Further studies by these researchers showed that wild raspberry extract produced after digestion, which is a source of natural polyphenols and flavonoids, has a distinct protective effect on the ACR-treated CaCo2 cells, as it inhibits the production of intracellular ROS and increases the mitochondrial membrane potential (MMP) and the level of glutathione (GSH) [46]. Similar antioxidative properties against ACR-induced oxidative damage of HepG2 cells are exhibited by blackberry extract after in vitro gastrointestinal digestion [47]. Further, Cao et al. [122] reported that curcumin reduces DNA damage in HepG2 cells through an antioxidant protective mechanism. In turn, Qu et al. [123] demonstrated that the jackfruit flake extract (JFE) and digestive products of jackfruit flake after gastrointestinal (JFG) reduced the cytotoxic effect of ACR on Caco-2 cells, increasing cell viability and alleviating mitochondrial disorders by reducing excessive ROS production. JFG also showed higher antioxidative properties and more effective protection than JFE. Similar results have been shown in studies on animal models. N-acetylcysteine, one of the major antioxidants in human and animal organisms, inhibits oxidative stress and limits the cytotoxic effect of ACR on the liver and the small and large intestine in rats by increasing the GST activity and decreasing the MDA level at pharmacological doses [45]. A similar protective effect on ACR-induced small and large intestine damage in rats is shown by an increase in the GSH level and the total antioxidant status, which is shown by crocin [38]. It has also been shown that the carica papaya fruit aqueous extract has antioxidant and immunostimulatory properties, protecting the stomach, liver, and kidneys of rats against acrylamide-induced oxidative stress and immune status disorders [42]. Despite these numerous substances that limit the negative impact of ACR on the gastrointestinal tract, the study was conducted on cell lines and rodents under laboratory conditions. Further epidemiological studies are necessary to confirm that these substances also have a positive impact on the human body. Most natural substances examined so far lose their beneficial properties during thermal treatment, and ACR is formed during the thermal processing of the products at high temperatures, which significantly limits their usability in the production of ACR-rich products.

The use of ACR-decomposing bacteria is the next step towards reducing ACR bioavailability in the gastrointestinal tract. Lactic acid bacteria were shown to have the ability to reduce the ACR level in food products [24,78,124,125,126]. The highest effectiveness in decreasing the ACR level has been shown for Streptococcus lutetiensis, Lactobacillus plantarum, and Lactobacillus casei Shirota, both under laboratory conditions and in an environment that mimics the conditions in the stomach and in the intestine [78,124,125]. Further studies have shown that bacterial consortia containing probiotic bacteria under simulated gastrointestinal conditions reduce ACR bioavailability much more effectively than bacterial monocultures [126]. It is expected that further studies will help to select microorganisms effective in ACR detoxication that will be used in food production and will allow for the production of new functional products. 

## 7. Conclusions

The studies conducted to date have shown that the gastrointestinal tract, as the first line of contact with ACR in food, is directly exposed to its toxic effects. ACR disrupts the integrity of tight junctions, leading to barrier dysfunction and intestine damage. ACR modifies intestinal enzyme activity, disrupts the function of the local immune system and of the enteric nervous system, and affects the gut microbiome, leading to alimentary tract dysfunction. The main mechanism of the ACR cytotoxic effect on the gastrointestinal tract involves disruption of the antioxidative balance and oxidative stress induction. Although the carcinogenic effect of ACR was confirmed only in rodent studies, the threat arising from ACR ingestion to human health cannot be totally excluded. A range of recommendations and strategies for decreasing the ACR level in food products and limiting its in vivo toxicity have been developed, and great interest has recently been shown in probiotic microorganisms. Further cooperation between food producers, regulatory bodies, researchers, and consumers is necessary to limit ACR consumption and its negative impact on the human body.

## Figures and Tables

**Table 2:** Microorganisms able to degrade ACR.

Microogranism	Enzyme Name (s)	Reference
*Arthrobacter* sp. DBV1	Acrylamidase	[59]
*Bacillus cereus* strain DRY135	Aliphatic amidase	[60]
*Bacillus clausii* strain 1779	Aliphatic amidase	[61]
*Burkholderia* sp. *strain DR.Y27*	Amidases of short-chain aliphatic	[62]
*Cupriavidus oxalaticus ICTDB921*	Acrylamidase	[63]
*Delftia tsuruhatensis ZJB-05174*	R-Enantio-selective amidase	[64]
*Enterobacter aerogenes*	Aliphatic amidase	[65]
*Helicobacter pylori 26695*	Amidase AmiE	[66]
Moraxiella osloensis MSU11	Aliphatic amidase	[67]
*Nocardia globerula* NHB-2	Amidase	[68]
*Pseudomonas aeruginosa*	Aliphatic amidase	[69]
*Pseudomonas aeruginosa* BAC-6	Aliphatic amidase	[70]
*Pseudomonas putida MTCC 6809*	Extracellular amidase	[71]
*Rodococcus* sp.	Aliphatic amidase	[72]
*Stenotrophomonas acidaminiphila* MSU12	Acrylamidase	[73]

**Table 3:** Effect of ACR administration on the ENS neurons.

Part of GI Tract	Species	Dose of ACR and Time of Exposure	Effect	References
duodenum	guinea pigs	3 mg/kg of b.w./day for last 35 days of gestation	increased surface area and diameter of Auerbach’s and Meissner’s plexuses	[54]
jejunum	mice	0.5 mg/kg of b.w./day for 90 days	increased intensity of neurofilament staining, circularity of the nerve ganglia, and the neurofilament integrated density and the cross-sectional area of the nerve ganglion	[37]
ileum	rats	50 mg/kg of b.w./day for 10 days	decrease in the density of catecholamine-containing nerve fibres and tissue content of noradrenaline in the myenteric plexus, decrease in tissue content and immunoreactivity of CGRP, an increase in VIP immunoreactivity in the myenteric plexus, decrease in CGRP immunoreactivity and an increase in VIP and NPY immunoreactivity in the submucous plexus	[88]
stomach	pig	0.5 and 5 μg/kg of b.w./day for 28 days	Low dose: an increase in the population of neurons immunoreactive to GAL, CART, VAChT, VIP, and SP in submucosal plexuses in the corpus and an increase in the number of GAL-, nNOS-, and VIP-immunoreactive in myenteric plexuses neurons in the cardia, corpus and pylorus, VAChT-, and SP-immunoreactive in the corpus and pylorus, and in the case of CART the changes were significant only in the corpus, and CGRP only in the pylorus. High dose: an increase in the number of GAL-, CART-, VAChT-, VIP-, SP-, and nNOS- immunopositive neurons in submucosal plexuses in the corpus of the stomach and an increase in the number of myenteric plexuses neurons immunopositive for GAL, CART, VAChT, VIP, SP, CGRP, and nNOS in the cardia, corpus, and pylorus.	[89,90,91]
duodenum	pig	0.5 and 5 μg/kg of b.w./day for 28 days	Low dose: an increased population of PACAP-, CART-, CGRP-, VAChT-, GAL- and SP- positive neurons in the myenteric plexuses; an increase in the number of VAChT-, GAL- and SP-positive neurons in the OSP; increased the population of CGRP-, VAChT-, VIP-, GAL-, and SP-immunoreactive neurons in the ISP. High dose: an increased population of PACAP-, CART-, CGRP-, VAChT-, VIP-, nNOS-, GAL-, and SP-immunoreactive neurons in the MP, OSP and ISP.	[86,92,93,94]
jejunum	pig	0.5 and 5 μg/kg of b.w./day for 28 days	Low dose: increased immunoreactivity with respect to PACAP, CART, CGRP, VAChT, GAL, and SP in the MP; an increased number of PACAP-immunoreactive neurons in the OSP; an increase in the number of PACAP-, VIP-, and GAL-immunoreactive and a decrease in the nNOS- positive neurons in the ISP. High dose: an increase in the population of PACAP-, CART-, CGRP-, VAChT-, VIP-, GAL-, and SP-immunoreactive and a decrease in that of nNOS- positive neurons in the MP, OSP, and ISP.	[86,92,93,94,95]
ileum	pig	0.5 and 5 μg/kg of b.w./day for 28 days	Low dose: an increase in the number of CART-, CGRP-, VIP-, GAL-, and SP-immunopositive and a decrease in the number of nNOS- positive in the MP; an increase in immunoreactivity with respect to VAChT, VIP, and SP and a decrease in the number of nNOS- immunoreactive neurons in the OSP; increased number of CGRP-, VAChT-, VIP-, and GAL-immunoreactive neurons the ISP. High dose: an increase in the number of PACAP-, CART-, CGRP-, VAChT-, VIP-, GAL-, and SP- immunoreactive and decreased the number of nNOS- positive neurons in the MP, OSP, and ISP.	[39,86,92,93,94]

Neuroactive substances: CART—cocaine- and amphetamine-regulated transcript peptide, CGRP—calcitonin gene-related peptide, GAL—galanin, nNOS—neuronal nitric oxide synthase, NPY—neuropeptide Y, PACAP—pituitary adenylate cyclase-activating polypeptide, VIP—vasoactive intestinal polypeptide, VAChT—vesicular acetylcholine transporter. Enteric plexuses: MP—myenteric plexus, OSP—outer submucous plexus, ISP—inner submucous plexus.

**Table 4:** The impact of dietary exposure to ACR on the development of cancers in the GI tract of rodents.

Part of GI Tract	Species	Dose of ACR and Time of Exposure	Effect	References
colon	rats	ACR in dose 10 mg/kg of b.w./day + diets supplemented with 10% corn oil for 8 weeks and then only 10% corn oil for 48 weeks	colonic aberrant crypt foci and colon cancer invasion; apoptosis was decreased and cell proliferation was increased in colonic mucosa; mitochondrial wt p53 was significantly inhibited through decreased mitochondrial localization of wt p53 and increased cytosolic p53, resulting in the up-regulation of Bcl-2 and the down-regulation of Bax in the mitochondria, inhibition of the release of cytochrome-c from the mitochondria into the cytosol and protein level of caspase-3	[103]
colon	rats	ACR in dose 5 mg/kg of b.w./day + diet supplemented with 10% corn, olive, beef, or fish oil for 8 weeks and then diets supplemented with 10% oil for other 40 weeks	ACR and diet with corn oil and beef tallow enhanced colonic aberrant crypt foci formation, increased BrdU incorporation, expression of cytosolic beta-catenin and cyclin D1 and decreased apoptosis in the colon mucosa; ACR and diet with beef tallow increased expressions of Wnt2 and Wnt3; ACR and diet with corn oil increased expressions of Wnt5a; ACR and diet with olive and fish oil weakened the colonic aberrant crypt foci formation	[104]
colon	rats	subcutaneous injection of azoxymethane and ACR at 0, 5, 10 or 50 mg/kg diet and low fat (7% corn oil) or high fat (23.9% corn oil) for 8 weeks	ACR does not increase the risk of developing azoxymethane-induced precancerous lesions of the colon in rats the highest tested dose of ACR (50 mg/kg diet) had significantly lower total colonic aberrant crypt foci and lower large colonic aberrant crypt foci compared with their respective controls	[105]
	rats	0.5, 1.0 or 2.0 mg/kg diet for 2 weeks and then subcutaneous injection with azoxymethane once a week for 2 weeks and diet with ACR for 20 weeks	ACR alone at doses corresponding to actual levels in food does not induce cancer in rats, but it can intensify azoxymethane-induced cancers when administered at larger doses	[106]
colon	mice	subcutaneous injection in the right shank with HT-29 human colon adenocarcinoma cells and after 3 weeks diet with ACR (0.5, 1.0 or 2.0 mg/kg diet) for 4 weeks	no differences in the growth of human colon tumour xenografts between acrylamide-treated and control mice	[106]
colon	mice	ACR in a dose 0.1 mg/kg of b.w./day for 4 weeks	genes implicated in RNA metabolism, processing and formation of the ribosomal subunits and protein translation and metabolism are upregulated in ACR-exposed colon tissue	[107]
